# An Alternative Serological Measure for Assessing Foot-and-Mouth Disease Vaccine Efficacy against Homologous and Heterologous Viral Challenges in Pigs

**DOI:** 10.3390/vaccines12010010

**Published:** 2023-12-21

**Authors:** Jaejo Kim, Seung Heon Lee, Ha-Hyun Kim, Sung-Ho Shin, Sang-Hyun Park, Jong-Hyeon Park, Choi-Kyu Park

**Affiliations:** 1 Animal and Plant Quarantine Agency, 177 Hyeoksin 8-ro, Gimcheon City 39660, Gyeongsangbuk-do, Republic of Korea; jkim1209@korea.kr (J.K.); seungheon0117@korea.kr (S.H.L.); hahy1202@naver.com (H.-H.K.); imshin121@korea.kr (S.-H.S.); shpark0205@korea.kr (S.-H.P.); 2 College of Veterinary Medicine & Animal Disease Intervention Center, Kyungpook National University, Daegu 41566, Republic of Korea

**Keywords:** foot-and-mouth disease, vaccination, indirect potency test, virus neutralization antibody, estimating the protection status

## Abstract

To analyze the relationship between homologous and heterologous serological titers of immunized pigs and their protection statuses against FMD virus challenges, in the present study, the correlation between the virus neutralization titers at 21 and 28 dpv and the protection statuses at 28 dpv against challenge with FMD virus was analyzed using data sets comprising five different combinations of homologous or heterologous challenge experiments in pigs vaccinated with type O (*n* = 96), A (*n* = 69), and Asia 1 (*n* = 74). As a result, the experiments were divided into three groups (21D-1, 21D-2, and 21D-3) in the 21-dpv model and two groups (28D-1 and 28D-2) in the 28-dpv model. Each response curve of groups 21D-1 and 21D-2 in the 21-dpv model was very similar to each curve of groups 28D-1 and 28D-2 in the 28-dpv model, respectively, even though there was an exceptional extra group (21D-3) in the 21-dpv model. The average titers estimating 0.75 probability of protection ranged from 1.06 to 1.62 log_10_ in the 21-dpv model and from 1.26 to 1.64 log_10_ in the 28-dpv model. In summary, we demonstrated that the serological method is useful for predicting the homologous and heterologous protection statuses of vaccinated pigs.

## 1. Introduction

Foot-and-mouth disease (FMD) is an economically important vesicular disease found in cloven-hoofed animal species, including cattle, pigs, sheep, and goats [1]. The causative agent, foot-and-mouth disease virus (FMDV), belongs to the genus *Aphthovirus*, family *Picornaviridae.* There are seven immunologically distinct serotypes of FMDV: O, A, C, Asia 1, SAT 1, SAT 2, and SAT 3 [2]. Given the fast transmission among hosts and the economic consequences of FMD, vaccination is widely used to contain and/or eradicate the disease in regions of the world where FMD is endemic. However, due to the narrow spectrum of cross-immunity between strains even of the same serotype, the selection of the proper vaccine strain is considered a critical point to control invasive FMD outbreaks [3]. Among the methods for the selection of FMD vaccine strains, in vitro vaccine matching tests can be considered to measure the serological relatedness between field isolates and a vaccine strain with a bovine vaccinated serum (r_1_ value). If the r_1_ value that is derived by dividing the heterologous serological titer by the homologous serological titer is greater than 0.3 in the case of neutralization, the relatedness between the field isolate and vaccine strain is considered sufficiently acceptable such that the vaccine containing the vaccine strain might be adequate to confer protective immunity against challenges with the field virus [4,5]. Nevertheless, recent studies have demonstrated that a higher potency vaccine could induce a wider spectrum of cross-immunity and earlier protection [6,7,8,9].

The efficacy of the FMD vaccine has been assessed via vaccination challenge tests in cattle using standardized in vivo FMD vaccine potency estimations, such as the PD_50_ (50% protective dose) and the PGP (protection against generalized foot infection) test [4]. However, in vivo challenge tests have practical and logistical problems [10,11], and several disadvantages from the perspective of animal welfare and biosafety. In these regards, alternative methods for assessing FMD vaccine efficacy are allowed if statistical correlations between the alternatives and the in vivo challenge test in the target animal species are established [4]. Therefore, many studies have developed or demonstrated statistically validated alternative tests using serological measures for estimating the potency of vaccines, predicting cross-protection and reducing animal experiments in cattle [12,13,14,15,16,17,18,19,20], as cattle are the main animals susceptible to vaccination with FMD vaccines in most countries. However, these approaches based on efficacy studies in pigs have seldom been reported in recent years, although several studies were conducted a few decades ago [21]. In addition, as FMD outbreaks intermittently occurred in Korea despite massive FMD vaccination campaigns, questions were eventually raised about the potential protection by vaccine-induced antibody titers against the outbreak virus due to a perception that pigs were not well protected by the FMD vaccine. Therefore, it has been speculated that statistical correlations between potential alternative serological measurements and the protection against FMD challenges in pigs need to be studied using currently adopted serological systems because pigs are the major susceptible animals to FMDV in Korea.

Among these indirect serological assessments is the expected percentage of protection (EPP), a potency test that South American researchers devised based on the correlative comparison of an enzyme-linked immunosorbent assay (ELISA) of sera collected at 60 days post vaccination (dpv) with the corresponding protection status as a result of a challenge with virulent FMDV at 90 dpv in PGP experiments [14,18]. In addition, pass-level studies on the PD_50_ have often been conducted with sets of data comprising the results of virus neutralization test (VNT) titers of sera collected at 21 dpv with the corresponding protection status resulting from a challenge with virulent FMDV at 21 dpv [11,16,19,20]. However, the World Organisation for Animal Health (WOAH) terrestrial manual indicates that the PD_50_ of oil-adjuvanted FMD vaccines are measured by challenge with the virulent FMDV at up to 28 dpv [4], and many efficacy tests in pigs have tended to be evaluated by challenge with FMDV at 28 dpv [22,23,24]. This discrepancy needs to be investigated regarding the pass-levels of the efficacy test for the Korean national standard, which are serological titers at 21 days after a full-dose immunization.

In this study, we established an indirect assessment model for the protection probability of FMD vaccines against serotypes O, A, and Asia 1, based on a statistical correlation between the protection conferred against virus challenges and the serological response in vaccinated pigs. We studied how two different sets of VNT titer results at 21 and 28 dpv could influence the development of the model of protection probability for FMD vaccines.

## 2. Materials and Methods

### 2.1. Cells and Viruses

Baby hamster kidney (BHK-21) and porcine kidney (LFBK) cells were used to culture FMDV in Dulbecco’s modified Eagle’s medium. The LFBK cells were kindly provided by the Plum Island Animal Disease Center (New York, NY, USA). The BHK-21 suspension cells were cultured in ProVERO-1 serum-free medium (Lonza Bioscience, Basel, Switzerland). Because we were interested in protective efficacies of vaccine strain candidates against the FMDV isolates causing outbreaks in Korea, the FMDV O/SKR/Jincheon/2014 (O/Jincheon, O/SEA/Mya-98 lineage), A/SKR/Pocheon/2010 (A/Pocheon, A/ASIA/Sea-97 G1 lineage), and A/SKR/Yeoncheon/2017 (A/Yeoncheon, A/ASIA/Sea-97 G2 lineage) were prepared for vaccine strains, challenges, and VNTs. And Asia-1/Shamir/ISR/89 (Asia-1/Shamir) strains were also prepared for vaccine strains, challenges, and VNTs, because outbreaks of type Asia-1 viruses were often reported in Pool 1 area [25].

### 2.2. Preparation of Vaccines

Four experimental monovalent vaccines (the O/Jincheon, A/Pocheon, A/Yeoncheon, and Asia-1/Shamir vaccines) and one commercial monovalent vaccine (O/Primorsky, O/SEA/Mya-98 lineage) were administered to pigs for efficacy testing. The experimental vaccines were formulated with each of the vaccine antigens and Montanide ISA 206 adjuvant (Seppic, Paris, France) as a double oil-emulsion, water-in-oil-in-water (W/O/W) formulation [26]. Briefly, to prepare the four experimental vaccines, the inactivated 146S antigens of FMD viruses were diluted with Tris-NaCl buffer (pH 7.6) and then added to Montanide ISA 206, 10% aluminum hydroxide gel, and 0.5 mg/2 mL saponin. The mixture was stirred at 300 rpm for 10 min at 30 °C in a water incubator to formulate a W/O/W emulsion.

### 2.3. Vaccine Efficacy Study and Challenge Protocol

Animal experiments were performed in a biosafety level 3 (BSL3) containment facility at the Animal and Plant Quarantine Agency (APQA). All experiments were followed by protocols were approved by the Institutional Animal Care and Use Committee (IACUC) of the APQA of Korea (IACUC Nos. 2018-407, 2019-435, and 2020-376). All animals were acclimated for at least 1 week before being subjected to experimentation. All experimental animals were cared for according to the animal management guidelines of the APQA.

Two hundred and seventy-four FMD-free, crossbred pigs (8–10 weeks of age) were used in the experiment to evaluate the efficacy of the five FMD vaccines. Pigs with 0.9 or less preexisting VNT titers against the challenge viruses were selected for efficacy tests. Twenty-nine and sixty-seven pigs were vaccinated with the O/Jincheon and O/Primorsky vaccines, respectively. Fifteen and fifty-four pigs were vaccinated with the A/Yeoncheon and A/Pocheon vaccines, respectively. Seventy-four pigs were vaccinated with the Asia-1/Shamir vaccine. To measure the serological titers after vaccination, sera were collected at 0, 21, and 28 days after vaccination. One serum sample from O/Primorsky-vaccinated pigs and sixteen serum samples from Asia-1/Shamir-vaccinated pigs at 21 dpv were mistakenly not collected.

At 28 dpv, a FMDV challenge was performed with a homologous or heterologous virus strain to the vaccine strain by intradermal inoculation into two sites on the upper surface of the tongue. In brief, according to the applied vaccine antigen serotypes, the vaccinated and control pigs were challenged with the same serotype of challenge viruses by intradermal inoculation with 0.2 mL of challenge virus containing 10^5.7^ TCID_50_/200 µL of O/Jincheon, 10^5.3^ TCID_50_/200 µL of A/Yeoncheon, or 10^6.3^ TCID_50_/200 µL of Asia-1/Shamir virus, which were determined to be 1000 × 50% porcine infectious dose (PID_50_) by pig challenge tests with pig-derived viruses. After the challenge, the titers of the viruses were confirmed by TCID_50_ in LFBK cells. The clinical scores of the infected pigs were collected daily and recorded using a scoring system described previously [27]. Through daily observation, the animals that showed clinical manifestations of typical FMD signs were immediately separated from other animals that did not show any clinical signs. After the FMDV challenge, sera were collected on all sequential days beginning at 0 days post challenge (dpc) to 7 dpc. Animals that developed typical FMD lesions, such as vesicle, at sites other than the tongue, such as the lips, gums, snout, and feet, were considered unprotected animals.

### 2.4. Serological Assays

The VNT titers against the FMDV strains in the serum samples were measured following the Manual of Diagnostic Tests and Vaccines for Terrestrial Animals of the WOAH [4]. Briefly, sera inactivated at 56 °C for 30 min were diluted serially and incubated with 100 TCID_50_ of the appropriate FMDVs at 37 °C for 1 h. O/Jincheon virus was added to sera from the efficacy test of the O/Jincheon and O/Primorsky vaccines; A/Yeoncheon virus was added to sera from the efficacy test of the A/Yeoncheon and Pocheon vaccines; and Asia-1/Shamir virus was added to sera from the Asia-1/Shamir vaccine efficacy test. LFBK cells were added to the microplate and incubated at 37 °C for 42–72 h to identify the cytopathic effect. The VNT titers were calculated as the log_10_ of the reciprocal serum dilution found to inhibit cytopathic effects.

### 2.5. Statistical Analysis

To find the correlation between the protection result and the serological titer, logistic regression was used as the statistical model, which was generated in the R program (version 4.3.0; http://www.r-project.org/, accessed on 1 April 2023) using the R2jags and coda packages. The binary protection variables (protected/not protected against challenge) were paired with the log-individual serological titers to construct the data set in the model. The expected probability of protection against the challenge (*p_i_*) for the log_10_ serum titer of an individual animal (*t_i_*) is given by log [*p_i_*/(1 − *p_i_*)] = *a* + *b*log_10_ *t_i_*. In the model, *a* and *b* are the intercept and slope parameters, respectively. Because various serotypes and strains could influence the level of protection at a given titer, the slope and intercept parameters were estimated in a Bayesian framework, considering various sets of combinations among serotypes or strains using a previously described procedure [20]. Briefly, several separate and combined sets of variation among strains and serotypes were considered for slope and intercept parameters. Grouping allocations were considered based on a post hoc comparison of estimated intercepts and slopes. The considered models are described in Table S1. For the estimation in a Bayesian framework, a Bernoulli likelihood was assumed for the binary protection status of the data. The deviance information criterion (DIC) was used to compare the fit of different models for serotype and strain variation in parameters [28].

Using a receiver operating characteristic (ROC) analysis [29], a cutoff VNT titer was calculated at the point of the highest prediction accuracy (ACC). Using this cutoff VNT titer as a criterion of protection against the challenge, the expected protection for the individual serum titer was predicted to analyze the diagnostic performance of the model in real values.

## 3. Results

### 3.1. In Vivo Efficacy Results

The results of the vaccination-challenge efficacy trials conducted in the APQA for three different serotypes are summarized in Table 1 and Data S1. In the efficacy trials, the VNT titers against the challenge strain varied widely due to the various amounts of purified FMD 146S antigen in the applied vaccines. The mean VNT titers at 28 dpv in the O/Jincheon and A/Yeoncheon groups were below the 1.0 log_10_ titer, showing 31.0 and 26.7% protection rates, respectively. In contrast, the mean VNT titers at 28 dpv in the O/Primorsky and Asia-1/Shamir groups were above the 1.30 log_10_ titer, showing 73.1 and 73.0% overall protection rates, respectively. The A/Pocheon-vaccinated groups had a 1.17 log_10_ mean VNT titer, and 64.8% of the vaccinated animals were protected. As the VNT titers increased sharply after the challenge, mean VNT titers against the challenge viruses of more than 2.15 log_10_ were found in all groups at 7 dpc.

**Figure 1:**
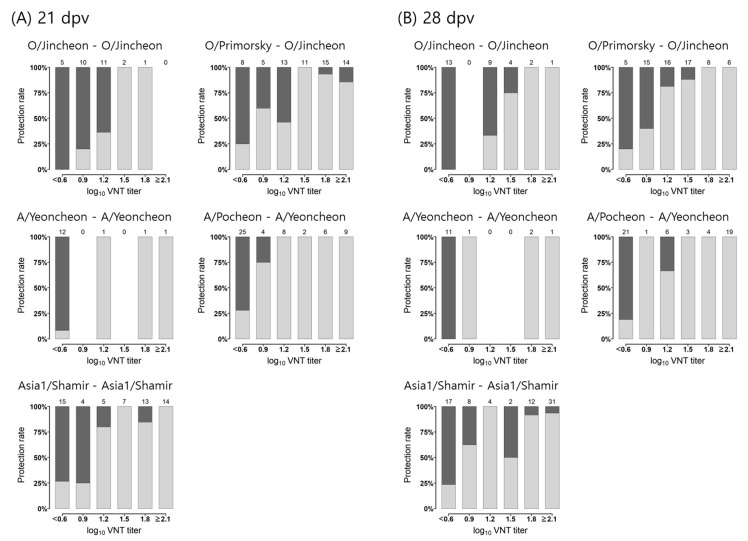
Observed protection rates based on levels of log_10_ virus neutralized test (VNT) titers following the challenge in the vaccine-challenge studies. (**A**) The protection rate is depicted by a relationship with the VNT titer at 21 dpv, and (**B**) the protection rate is described according to each VNT titer at 28 dpv. The vaccine and challenge strains in each study are indicated as hyphenated words at the top of each plot. Each bar represents a group of animals sharing the indicated titer ±0.15 (reciprocal log_10_). The numbers of animals with each titer level are indicated at the top of each bar. Gray part of bars, protected; black part of bars, not protected.

The observed protection rates against the challenge viruses based on the levels of VNT titers are illustrated in Figure 1. According to the observed protection statuses at challenge time for each 21 and 28-dpv VNT titer, protection rates tended to be higher as VNT titers increased. Twenty-eight percent or less protection rates were observed in animals with less than 0.6 log_10_ VNT titer in each vaccine-challenge study. More than 84% protection rates were observed in animals with more than 1.65 log_10_ VNT titers.

### 3.2. Estimation of Protection Probability to VNT Titer

**Figure 2:**
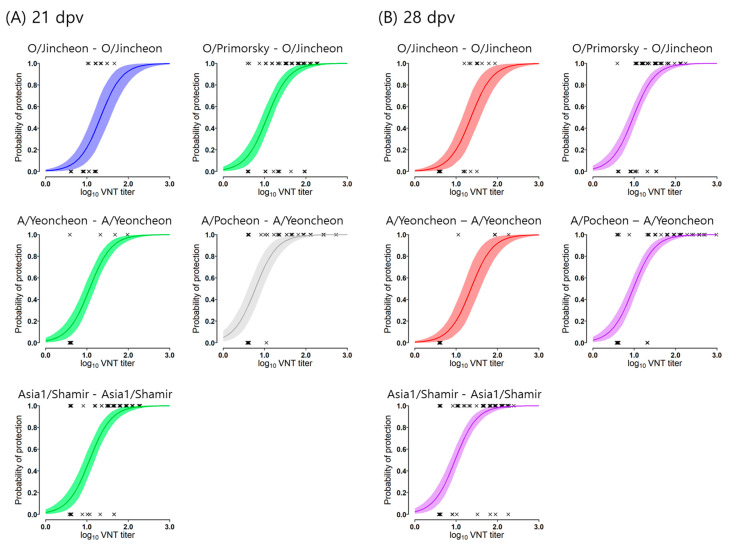
Estimated probability of protection for virus neutralization tests (VNT) against the challenge strains. (**A**) The plots of the probability of protection are depicted according to each VNT titer at 21 dpv, and (**B**) the plots of probability of protection are depicted according to each VNT titer at 28 dpv. Color indicates individual groups: three groups in 21-dpv models, group 21D-1 (blue), group 21D-2 (green), and group 21D-3 (gray); two groups in 28-dpv models, group 28D-1 (red), and group 28D-2 (purple).

By analyzing the logistic regression models in a Bayesian framework, the preferred models for 21- and 28-dpv titers were determined (Table S1). Although the 28-dpv model, which contained five different groups of strains, was found to be the best based on comparisons of the respective DICs, the model in which the intercepts fell into two groups and shared a slope in common was preferred, because its DIC was very similar to the model comprising five independent groups and because it was simpler than most other models. Therefore, the models for 21- and 28-dpv in which the intercepts were different across groups and the slope was common were preferred (Figure 2). In the 21-dpv model, the correlative relationships of the probability of protection with VNT titers were defined as experiments and were divided into three groups: group 21D-1 comprising O/Jincheon-O/Jincheon; group 21D-2 comprising O/Primorsky-O/Jincheon, A/Yeoncheon-A/Yeoncheon, and Asia1/Shamir-Asia1/Shamir; and group 21D-3 comprising A/Pocheon-A/Yeoncheon trials. In the 28-dpv model, two separate groups of trials were identified as models for predicting the probability of protection with VNT titers: group 28D-1 comprising the O/Jincheon-O/Jincheon and A/Yeoncheon-A/Yeoncheon trials and group 28D-2 comprising the O/Primorsky-O/Jincheon, A/Pocheon-A/Yeoncheon, and Asia1/Shamir-Asia1/Shamir trials. The estimated parameters for both models are described in Table S2.

**Figure 3:**
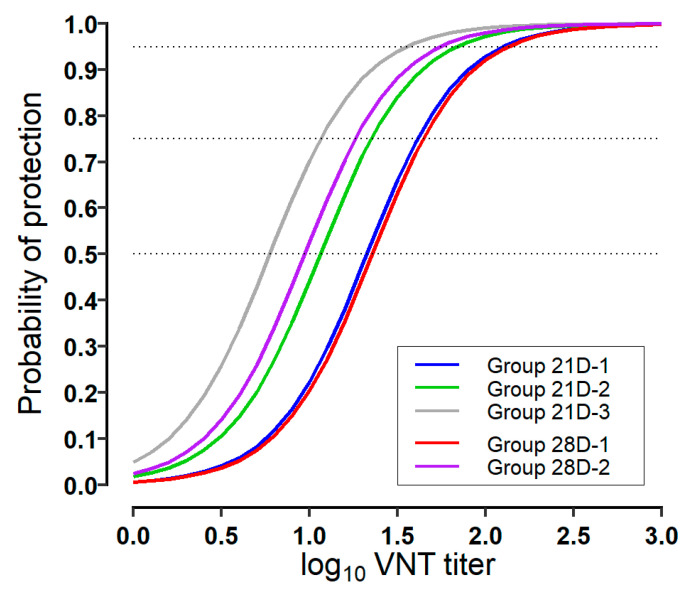
Logistic models of the probability of protection following the challenge for the log_10_ VNT titer. The plot shows the posterior median for the probability of protection for group 21D-1, group 21D-2, and group 21D-3 in the 21-dpv model, and group 28D-1 and 28D-2 in the 28-dpv model.

The response curves of the models of different groups in each model are shown in comparison with one another in Figure 3. Each response curve of group 21D-1 and group 21D-2 in the 21-dpv model is very similar to those of group 28D-1 and group 28D-2 in the 28-dpv model, respectively. The probability of protection tends to correlate to higher VNT titer in order of the first and second groups in each model. The curve of group 21D-3 in the 21-dpv model corresponds most effectively to the VNT titer. The estimated VNT titers for protection probabilities based on the constructed models are described in Table 2. The estimated average VNT titers corresponding to 75% protection were 1.62, 1.35, and 1.06 log_10_ VNT titers in groups 21D-1, 21D-2, and 21D-3 of the 21-dpv model, respectively. The estimated average VNT titers corresponding to 75% protection were 1.34 and 1.26 log_10_ VNT titers in groups 28D-1 and 28D-2 of the 28-dpv model, respectively.

### 3.3. The Characteristics of the Models

**Figure 4:**
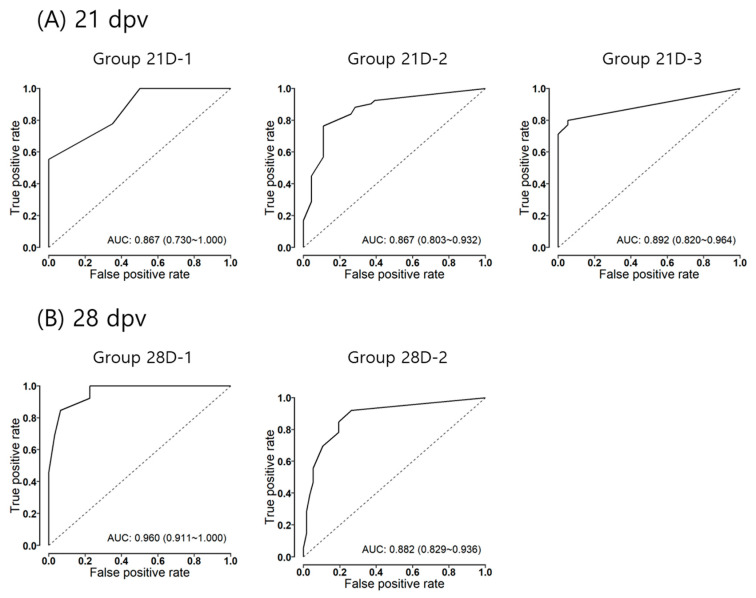
The ROC curves for the diagnostic performance of the indirect FMD vaccine potency test based on the VNT logistic regression models. (**A**) The plots of the ROC curves are depicted for different groups in the 21 dpv model, and (**B**) the plots of the ROC curves are depicted for different groups in the 28 dpv model. The AUC (area under the curve) of each model is indicated with a 95% confidence interval over the x-axis of each plot.

The results of the ROC analysis of each group in the 21- and 28-dpv models are illustrated in Figure 4. The mean values of the area under the curve (AUC) in all groups were more than 0.867 and 0.882 in the 21- and 28-dpv models, respectively. The cutoff VNT titers of protection were determined for each model using ROC analysis (Table 3). The cutoff VNT titers of protection of group 21D-1, group 21D-2, and group 21D-3 in the 21-dpv model were 1.27. 1.43, and 0.75 log_10_, respectively. The cutoff VNT titers of group 28D-1 and group 28D-2 in the 28-dpv model were 1.27 and 0.97 log_10_, respectively. The sensitivities of the groups in the 28-dpv models were 84.6% or greater, but those in the 21-dpv models ranged from 55.6% to 80.0%. The specificities in the 21-dpv models were greater than 89%, although the specificities in the 28-dpv models were greater than 73.7%. Although the ACCs of all groups in each model reached 80.6 or greater, the ACCs in the 28-dpv model were higher than those in the 21-dpv model.

## 4. Discussion

While highly effective for measuring protection, in vivo FMD vaccine efficacy tests are rarely performed due to the expense, time, and the need for high containment bio-security animal facilities to prevent the environment from subsequent infection. Therefore, sample size imbalances of in vivo FMD vaccine efficacy data are often observed in statistical approach studies [16,20]. In this study, the Bayesian framework was used to draw the reliable statistical inference from a limited number of samples in groups due to limited resources and time, like the study by Gubbins et al. [20]. In the present study, the correlation between the VNT titer and protection status against the challenge with FMDV was analyzed using data sets comprising five different combinations of homologous or heterologous vaccination challenge experiments in pigs. According to the results of the 21-dpv and 28-dpv models, we speculate that universal models with common parameters cannot be established to predict homologous and heterologous efficacy tests, as was seen in a study by Gubbins et al. [20]. In summary, the experiments are divided into three groups in the 21-dpv model and two groups in the 28-dpv model (Figure 2). Each response curve of groups 21D-1 and 21D-2 in the 21-dpv model was very similar to each curve of groups 28D-1 and 28D-2 in the 28-dpv model, respectively, even though there was an exceptional extra group in the 21-dpv model (Figure 3). Interestingly, a significant difference in the parameters between both models was shown in only the type A experiment. The type A homologous efficacy experiment (A/Yeoncheon-A/Yeoncheon), belonging to group 21D-2 in the 21-dpv model, correlated with group 28D-1 in the 28-dpv model, and the type A heterologous efficacy experiment (A/Pocheon-A/Yeoncheon), belonging to group 21D-3 in 21-dpv, correlated with group 28D-2 in the 28-dpv model. One finding related to this change was that the VNT titers induced by the A/Pocheon vaccine between 21 and 28 dpv changed the most significantly among the vaccine-induced VNT titers in this study. Because fluctuations in serological titers are often observed within approximately one month after FMD vaccination, the model should be carefully applied to predict the potency of the vaccine based on its original performance, even if the diagnostic test is guaranteed for its reproducibility as an indirect alternative.

According to Table 1, the mean VNT titers and protection rates against the homologous challenge seems to be lower than the result against the heterologous one in the efficacy groups. However, we had to prepare FMD vaccines formulated with various amounts of the purified FMD 146S antigen to induce a wide range of VN titers in immunized pigs. Reflecting on the antigen payloads in vaccines, it could be speculated that a greater amount of antigen in the vaccines may cause a significant effect in inducing a higher level of VNT and more protection, in the situation where homologous and heterologous strains may share common neutralization antigenic sites because both strains belong to the same lineage. But we have no direct evidence for this, because we did not prepare homologous and heterologous vaccines with a comparable payload of the 146S antigen.

Alternative serological methods based on the statistical correlation between serological titers and protection status against challenges with homologous viruses have often been devised to estimate the efficacy of FMD vaccines against homologous vaccine viruses in cattle. The statistical correlation between serological titers and protection status in porcine efficacy tests has also indicated that a mean VNT titer of 1.74 or higher equated to 62.5% protection in 92% cases of porcine efficacy tests challenged with the O1 BFS 1860 virus after vaccination with oil-emulsion FMD vaccines [21]. However, the current WOAH minimum potency level for FMD vaccines is three PD_50_. In South America, the liquid phase blocking ELISA titer for each FMD vaccine strain was predetermined for the assessment of the approval of the vaccine release test using cattle with a criterion of 75% EPP, which correlated with a PD_50_ value of 3 [13,18]. Conversely, the probabilities of protection for 3 and 6 PD_50_ of oil-emulsion FMD vaccines were estimated to be 0.71 and 0.81 in the bovine efficacy test, respectively [30]. Another study reported that the VNT titer corresponding to a 0.75 probability of protection in cattle ranged from 1.17 to 2.46 log_10_ [19]. In the present study, the VNT titers related to a 0.625 probability of protection in pigs ranged from 0.91 to 1.46 log_10_ in the 21-dpv model and from 1.10 to 1.49 log_10_ in the 28-dpv model. The VNT titers estimating 0.75 probability of protection ranged from 1.06 to 1.62 log_10_ in the 21-dpv model and from 1.26 to 1.64 log_10_ in the 28-dpv model. It seems that the results of each study are somewhat different from one other, probably because there were several differences in hosts, FMD vaccine formulations and challenge viruses, routes of challenge, and the amounts of challenge virus administered among these studies. Additionally, VNT is well known to have poor reproducibility depending on the laboratory. However, as indirect alternatives, serological approaches have been well established to predict the efficacy of FMD vaccines. Among serological tests, VNT is more easily applicable than ELISA for quantifying antibody titers induced by a variety of FMD strains.

In the diagnostic performance of the present study, the overall accuracies were over 80% in both models. The accuracy (86.7–90.9%) of the 28-dpv model was better than that (80.6–86.2%) of the 21-dpv model according to the cutoff VNT titer to predict protection status. In the 21-dpv model, the cutoff VNT titer to predict the protection status of group 21D-3 is 0.75 log_10_, which is below 0.9 log_10_, the minimum titer found in this study. The cutoff VNT titer of group 28D-2 in the 28-dpv model was 0.97 log_10_, which is very similar to the minimum titer found in this study. These low cutoff titers mean that most animals in these groups tended to be protected even if the serological titers were barely detected. Approximately 25% of animals that had less than 0.9 log_10_ VNT titer at 21 or 28 dpv were protected in these groups. This tendency toward a low cutoff is probably related to the resistance of the host against a certain FMD strain. It could also be inferred that there is another immune response, such as cell-mediated immunity, related to protection in pigs. However, to clarify these results, further study with a larger scale of experiments is necessary.

Although the results from both models are different from one other, both models showed significant correlations between serological titers and protection status. However, it seems that the 28-dpv model has a more significant correlation than the 21-dpv model, because the 28-dpv model has a lower DIC value than the 21-dpv model and because the AUCs of the 28-dpv model ranged from 0.882 to 0.960, while those of the 21-dpv model ranged from 0.867 to 0.892 using ROC analysis.

Pigs play an important role as amplifiers of FMDV, releasing 3000× more aerosol virus than cattle within 24 h, especially in intensive farming areas [31]. Therefore, porcine efficacy tests sometimes fail to show the relationship between the serological titer and protection status because of immense superinfection by amplifying aerosol virus after inappropriate challenges [21]. Therefore, several pig challenge methods were carefully considered for this study, although various challenge methods have already been used in pig efficacy tests, including heel bulb intradermal inoculation [22,32,33,34,35], intramuscular inoculation [24], intra-oropharyngeal inoculation [36], and contact challenge by introduction with infected donor [37,38]. Although heel bulb intradermal inoculation is considered the major challenge method due to its superior sensitivity in pigs [39], its application in pigs is labor-intensive and time-consuming [24]. Additionally, because oropharyngeal tonsil epithelium is considered the natural primary infection site of FMDV in the host [40], contact challenge or intra-oropharyngeal inoculation is preferred to investigate the infection dynamics, transmission between hosts, and the pathogenesis of FMD in pigs [41,42,43]. In this study, pig challenge methods were adapted from those of cattle in the WOAH manual based on a pilot study with O/Jincheon virus. The experiments were conducted to determine the PID_50_ in pigs with O/Jincheon, A/Yeoncheon, and Asia1/Shamir. The 1000 PID_50_ for the challenge was determined by referring to porcine challenge studies [24,44,45]. In each porcine efficacy experiment, all unvaccinated control animals developed clinical signs of FMD within four days after the challenge.

We conclusively demonstrated that the correlation between titers and the probability of protection against the challenge virus was significant in FMD-vaccinated pigs. The possibility of VNT titers being specific to the FMD strain in regard to the corresponding probability of protection was also demonstrated. Additionally, vaccination challenge experiments were categorized to build prediction models with common parameters. According to the present study, the serological titer at the time of challenge could more correctly predict a probability of protection. However, it could be concluded that the serological titers at an earlier time of challenge, such as 21 days, could be used to estimate the protection if the model is statistically demonstrated. This study has limitations in the diversity of vaccination challenge experiments using more various vaccines and challenge strains, and it does not have data related to PD_50_ in pigs. Therefore, further research based on more porcine vaccination challenge experiments under various conditions should be conducted to develop better models to predict cross-protection against various FMD virus strains.

## Figures and Tables

**Table 1:** Summary of the serological mean VNT titers against the challenge strains in animals and the protection status used in the vaccination and challenge tests.

Serotype	Vaccine Strain	Challenge Strain	No. of Challenged Animals	No. of Protected Animals	VNT Titer ± SD ^1^ (log 10)			
					21 dpv ^2^	28 dpv	4 dpc ^3^	7 dpc
O	O/Jincheon	O/Jincheon	29	9	0.96 ± 0.48 ^1^	0.78 ± 0.74	1.59 ± 0.39	2.44 ± 0.22
	O/Primorsky	O/Jincheon	67	49	1.42 ± 0.62	1.30 ± 0.50	1.53 ± 0.40	2.15 ± 0.42
A	A/Yeoncheon	A/Yeoncheon	15	4	0.33 ± 0.69	0.48 ± 0.86	0.74 ± 0.95	2.63 ± 0.57
	A/Pocheon	A/Yeoncheon	54	35	0.88 ± 0.90	1.17 ± 1.02	1.78 ± 0.78	2.24 ± 0.48
Asia-1	Asia-1/Shamir	Asia-1/Shamir	74	54	1.26 ± 0.81	1.39 ± 0.86	1.72 ± 0.59	2.16 ± 0.27

^1^ Mean VNT titer ± standard deviation; ^2^ days post vaccination; ^3^ days post challenge.

**Table 2:** Estimated VNT titers (log_10_) according to the probability of protection at which vaccinated pigs are protected against the challenge with FMDV.

Model	Group	Probability of Protection				
		0.50	0.625	0.75	0.85	0.95
21-dpv	21D-1	1.33 (1.09–1.56) ^1^	1.46 (1.23–1.72)	1.62 (1.38–1.92)	1.78 (1.54–2.14)	2.10 (1.82–2.60)
	21D-2	1.06 (0.93–1.20)	1.19 (1.06–1.33)	1.35 (1.22–1.51)	1.52 (1.37–1.69)	1.83 (1.66–2.13)
	21D-3	0.78 (0.59–0.97)	0.91 (0.73–1.12)	1.06 (0.88–1.29)	1.23 (1.04–1.53)	1.55 (1.32–1.94)
28-dpv	28D-1	1.36 (1.12–1.59)	1.49 (1.26–1.75)	1.64 (1.41–1.94)	1.81 (1.57–2.17)	2.12 (1.85–2.60)
	28D-2	0.97 (0.87–1.09)	1.10 (0.99–1.22)	1.26 (1.15–1.39)	1.42 (1.30–1.58)	1.74 (1.57–2.00)

^1^ Estimated log_10_ VNT titer (95% CI).

**Table 3:** The predicted cutoff of VNT titer (log_10_) determined using ROC analysis and following overall performance in each logistic model.

Model	Group	VNT Titer Cutoff	Probability of Protection (95% CI ^1^)	Sens. (%)	Spec. (%)	ACC (%)
21-dpv	21D-1	1.27	0.45 (0.24–0.65)	55.6	100.0	86.2
	21D-2	1.43	0.80 (0.71–0.88)	76.3	89.1	80.6
	21D-3	0.75	0.48 (0.30–0.65)	80.0	94.7	85.2
28-dpv	28D-1	1.27	0.42 (0.22–0.63)	84.6	93.5	90.9
	28D-2	0.97	0.50 (0.39–0.61)	92.0	73.7	86.7

^1^ 95% confidence interval; Sens.: sensitivity = true positive rate; Spec.: specificity = 1 − false negative rate; ACC: accuracy of predictions.

## Data Availability

All data generated and/or analyzed during this study are included in this manuscript. The raw data are available from the corresponding author on reasonable request, or by referring to Supplementary Materials.

## Supplementary Materials

The following supporting information can be downloaded at: https://www.mdpi.com/article/10.3390/vaccines12010010/s1, Data S1: The serology results and protection outcomes for each animal; Table S1: Deviance information criterion (DIC) determined in each model for the probability of protection; Table S2: Estimates for the intercepts and slopes in the models for the probability of protection after challenge with FMDV.
