# Supplementary material for: An Alternative Serological Measure for Assessing Foot-and-Mouth Disease Vaccine Efficacy against Homologous and Heterologous Viral Challenges in Pigs
Source: Vaccines (Basel). 2023 Dec 21;12(1):10. doi: 10.3390/vaccines12010010 (PMC10820997; doi:10.3390/vaccines12010010)
Supplement: Supplementary file 1 [file vaccines-12-00010-s001.zip › Table S1.pdf]

Table S1. Deviance information criterion (DIC) determined in each model for the probability of protection.

Intercept (a)[1]	Slope (b)[1]	DIC[2]	
		21-dpv	28-dpv
Common	Common	196.2	183.5
Common	Serotype	188.6	187.0
Common	Strain	189.6	185.3
Serotype	Common	190.4	186.6
Strain	Common	190.4	182.4
Group[3]	Common	**187.5**	**176.5**
Serotype	Serotype	190.8	184.2
Strain	Serotype	190.7	180.3
Serotype	Strain	192.2	182.1
Strain	Strain	188.1	175.2

[1] To set more unified models, various sets of parameters were considered based on different serotypes and strains.

[2] As a model with a lower DIC is preferred to one with higher DIC, the preferred models were considered among models with lower DICs. In 21-dpv, the model in bold was preferred because its DIC was lowest among the models. In 28-dpv, the model in bold was determined to be preferred because the difference in its DIC with the model of the lowest DIC, which is the strain-based prediction model, is less than two, and the experiments need to be combined into smaller groups for simpler explanation of the relationships of serological titer with protection status.

[3] In 21-dpv, the experiments are divided into three groups: group 21D-1 comprising O/Jincheon-O/Jincheon; group 21D-2 comprising O/Primorsky-O/Jincheon, A/Yeoncheon-A/Yeoncheon, and Asia1/Shamir-Asia1/Shamir; and group 21D-3 comprising A/Pocheon-A/Yeoncheon trials. In the 28-dpv model, two separate trials were identified as models to predict the probability of protection with VNT titers: Group 28D-1 comprised the O/Jincheon-O/Jincheon and A/Yeoncheon-A/Yeoncheon trials, and group 28D-2 comprised the O/Primorsky-O/Jincheon, A/Pocheon-A/Yeoncheon, and Asia1/Shamir-Asia1/Shamir trials. These allocations are based on a *post hoc* comparison of estimated intercepts in which the slope was common.
