# Supplementary material for: An Alternative Serological Measure for Assessing Foot-and-Mouth Disease Vaccine Efficacy against Homologous and Heterologous Viral Challenges in Pigs
Source: Vaccines (Basel). 2023 Dec 21;12(1):10. doi: 10.3390/vaccines12010010 (PMC10820997; doi:10.3390/vaccines12010010)
Supplement: Supplementary file 1 [file vaccines-12-00010-s001.zip › Table S2.pdf]

Table S2. Estimates for the intercepts and slopes in the models for the probability of protection after challenge with FMDV.

Model	Group	Intercept (*a*)	Slope (*b*)
21-dpv	21D-1	-5.07[1] (-6.57 --3.69)[2]	3.81 (2.88-4.85)
	21D-2	-4.05 (-5.34 - -2.81)	
	21D-3	-2.96 (-4.01 - -1.89)	
28-dpv	28D-1	-5.20 (-6.67 - -3.81)	3.83 (2.91-4.82)
	28D-2	-3.72 (-4.85 - -2.65)	

[1] Posterior median, [2] 95% confidence interval
